# Feeder-free yet still naïve: improved method for capturing human pluripotent stem cells

**DOI:** 10.1038/s44318-026-00713-3

**Published:** 2026-03-12

**Authors:** Masaki Yagi, Konrad Hochedlinger

**Affiliations:** 1 Department of Molecular Biology, Center for Regenerative Medicine, Krantz Family Center for Cancer Research, Massachusetts General Hospital, Boston, MA 02114 USA; 2 Department of Genetics, Harvard Medical School, Boston, MA 02115 USA; 3 Harvard Stem Cell Institute, Cambridge, MA 02138 USA; 4 Broad Institute of MIT and Harvard, Cambridge, MA 02142 USA

**Keywords:** Cell Adhesion, Polarity & Cytoskeleton, Methods & Resources, Stem Cells & Regenerative Medicine

## Abstract

Recent work reports serum-coating as a practical alternative for culturing hPSCs26 while preserving their defining molecular and functional properties.

## Teaser

Current human pluripotency models are hindered by the need for co-culture with little-defined mouse feeder cells. In this issue, Rossignoli et al (2026) demonstrate that naïve human pluripotent stem cells (hPSCs) can be stably maintained long-term on serum-coated substrates without feeder cells, retaining their transcriptional and functional properties, and offering a scalable, reproducible, and broadly accessible platform for studying early human development.

## From feeder to feeder-free: challenges in capturing human naive pluripotency

**Figure 1:**
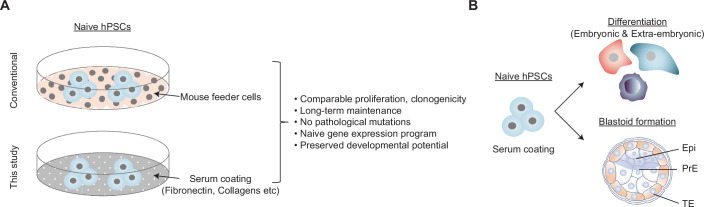
Serum coating as a feeder-free platform for modeling human naive pluripotency and early development. (**A**, **B**) Most current protocols for maintaining human naive pluripotency rely on mouse feeder cells. This study suggests that fibronectin and collagens deposited by serum coating enable the stable maintenance of naive hPSCs while preserving their genomic integrity, transcriptional program, and developmental potential, including in vitro lineage differentiation and blastoid formation. Epi epiblast, which gives rise to the three germ layers (endoderm, mesoderm, and endoderm), PrE primitive endoderm, which gives rise to the yolk sac, TE trophectoderm, which gives rise to the placenta.

Human pluripotent stem cells (hPSCs) provide a tractable and versatile system to study early human development and enable diverse applications, from disease modeling to regenerative medicine. Conventional hPSC cultures maintained in FGF and TGFβ resemble the post-implantation epiblast and are therefore considered to be in a primed state (Nichols and Smith, 2009). By contrast, naive hPSC cultures correspond more closely to the pre-implantation epiblast and display distinct transcriptional profiles, epigenetic landscapes, signaling dependencies, and metabolic features (Zhou et al, 2023). Accordingly, naive hPSCs exhibit a broader developmental potential than primed hPSCs, including the ability to give rise to extraembryonic lineages and self-organize into blastocyst-like structures termed blastoids (Kagawa et al, 2022; Liu et al, 2021; Yu et al, 2021). Given these unique properties, naive hPSCs represent a powerful platform for accessing early lineage specification and pre-implantation development in humans (Gafni et al, 2013; Takashima et al, 2014; Theunissen et al, 2014). Despite these obvious advantages, the routine use of naive hPSCs is limited by specific culture requirements. Unlike mouse PSCs, which can be stably maintained in chemically defined feeder-free conditions (Ying et al, 2008), conventional human naive hPSC protocols depend on mouse embryonic fibroblast (MEF) feeder layers to ensure robust self-renewal (Takashima et al, 2014; Theunissen et al, 2014). This dependence on MEFs introduces variability, requires continuous animal-derived products, and obscures the extracellular signals that directly support the naive state. Feeder-based culture also limits standardization across laboratories and complicates high-throughput applications, including comparative studies and mechanistic analyses. In this issue, Rossignoli et al (2026) sought to overcome these limitations by devising a simple yet robust method for the long-term, feeder-free culture of human naive hPSCs using serum-coated substrates (Fig. 1A). By revisiting a classical cell culture strategy with systematic validation, this study provides a practical alternative to feeder-based culture while preserving the defining molecular and functional properties of human naive pluripotency.

## Revisiting serum as a potential substrate for naive hPSCs

Serum has played an instrumental role in the development of cell culture protocols, long before extracellular matrix (ECM) components were molecularly defined. It is now well established that serum contains abundant ECM proteins, including fibronectin and vitronectin, which promote cell adhesion and proliferation (Chen et al, 2011). While various tissue culture coatings (e.g., Matrigel, Laminin-511) were previously reported to support the maintenance of primed hPSCs, it has remained unclear whether and to what extent serum-based substrates can maintain the naive state and support long-term self-renewal without compromising developmental potential and genomic integrity. This question is particularly relevant given the unique signaling requirements and epigenetic configuration of naive hPSCs. Rossignoli et al (2026) have addressed this question through a comprehensive and multi-laboratory study. Building off PXGL medium (Bredenkamp et al, 2019), a widely adopted condition for maintaining naive hPSCs, they systematically evaluated serum-coated substrates as a potential replacement for MEF feeders. By testing 30 independent serum batches across multiple naive hPSC lines and validating results in five independent laboratories, this study establishes that serum is as robust as, if not superior to, MEFs in maintaining naive hPSCs.

## The ‘voodoo’ behind serum

To gain insight into the molecular basis of serum-mediated hPSC support, the authors performed mass spectrometry analysis of different serum batches used to coat plastic dishes. Fibronectin and collagens were consistently detected across different batches, implicating classical ECM–integrin interactions in promoting hPSC adhesion and survival under naive conditions. These findings align with longstanding principles of cell biology but are notable in the context of human naive pluripotency, where the relative contribution of ECM cues versus feeder-derived signaling has been unclear. The data suggest that, at least in the context of PXGL medium, complex feeder-dependent signaling and physical cell-cell interactions may not be strictly required for maintenance of the human naive state, provided that appropriate ECM interactions are supplied. This raises important questions for future mechanistic investigation. For example, are fibronectin and collagens sufficient, or do additional serum-derived factors fine-tune naive cell identity? Can fully defined, recombinant ECM substrates recapitulate these effects, and how do integrin-mediated signals intersect with intracellular pathways that stabilize naive pluripotency? By eliminating mouse feeder cells, serum-coated systems now provide a cleaner and more tractable platform to address these fundamental questions.

## Preservation of naive identity and developmental potential

Whenever modified hPSC culture conditions are being introduced, it is imperative to consider potential changes to cellular state or developmental potential. Naive pluripotency is defined not only by morphology or a unique set of markers but also by coordinated transcriptional, epigenetic, and functional features. Remarkably, naive hPSCs maintained on serum-coated substrates closely resemble their feeder-cultured counterparts across several molecular and functional criteria. For example, gene expression analyses reveal highly similar naive-associated signatures, and cells display comparable proliferation rates, clonogenicity, and mutation frequencies over extended passages. Crucially, functional assays demonstrate preservation of developmental potential. Specifically, naive hPSCs cultured on serum-coated substrates efficiently differentiate into all three embryonic germ layers and retain competence to generate extraembryonic lineages, including trophectoderm (Fig. 1B). In addition, these cells can self-organize into blastoids with efficiencies comparable to those observed with feeder-based cultures (Fig. 1B). Because blastoid formation is highly sensitive to perturbations in the pluripotent state, this result provides compelling functional evidence that serum-coated substrates indeed support bona fide naive pluripotency.

## Implications and perspectives

Serum-coated substrates are inexpensive, scalable, and easy to implement, lowering technical barriers for laboratories adopting naive hPSC culture. Importantly, by eliminating mouse-derived feeder cells, serum-based substrates reduce the reliance on xenogeneic materials, which enhances reproducibility across experiments and laboratories and reduces concerns surrounding the potential immunogenicity and contamination of hPSC derivatives. This serum-based approach will facilitate applications that require standardization, including comparative studies between hPSCs, high-throughput screens, and efforts toward clinical translation. Beyond practical considerations, this study has broader conceptual implications. It challenges the assumption that human naive pluripotency intrinsically depends on complex and poorly defined feeder interactions. Simplified systems such as the one described here are likely to accelerate efforts to dissect the signaling and epigenetic mechanisms that stabilize naive pluripotency and govern lineage competence. For instance, it should be informative to compare serum batches competent for naive hPSC self-renewal with those unable to do so, in order to narrow down candidate molecules that support naive pluripotency. As naive hPSCs continue to serve as a foundation for modeling early human development, implantation, and lineage specification, the demand for robust and transparent culture systems will significantly increase. Serum-coated substrates offer a practical and effective approach while fully defined synthetic platforms continue to evolve (Bredenkamp et al, 2019; Takashima et al, 2014; Theunissen et al, 2014). Leveraging a longstanding cell culture method, Rossignoli et al (2026) advance the standardization of human naive hPSC culture across laboratories and strengthen the framework for investigating human pluripotency, differentiation, and development.
